# A recently quenched galaxy 700 million years after the Big Bang

**DOI:** 10.1038/s41586-024-07227-0

**Published:** 2024-03-06

**Authors:** Tobias J. Looser, Francesco D’Eugenio, Roberto Maiolino, Joris Witstok, Lester Sandles, Emma Curtis-Lake, Jacopo Chevallard, Sandro Tacchella, Benjamin D. Johnson, William M. Baker, Katherine A. Suess, Stefano Carniani, Pierre Ferruit, Santiago Arribas, Nina Bonaventura, Andrew J. Bunker, Alex J. Cameron, Stephane Charlot, Mirko Curti, Anna de Graaff, Michael V. Maseda, Tim Rawle, Hans-Walter Rix, Bruno Rodríguez Del Pino, Renske Smit, Hannah Übler, Chris Willott, Stacey Alberts, Eiichi Egami, Daniel J. Eisenstein, Ryan Endsley, Ryan Hausen, Marcia Rieke, Brant Robertson, Irene Shivaei, Christina C. Williams, Kristan Boyett, Zuyi Chen, Zhiyuan Ji, Gareth C. Jones, Nimisha Kumari, Erica Nelson, Michele Perna, Aayush Saxena, Jan Scholtz

**Affiliations:** 1 Kavli Institute for Cosmology, University of Cambridge, Cambridge, UK; 2 Cavendish Laboratory - Astrophysics Group, University of Cambridge, Cambridge, UK; 3 Department of Physics and Astronomy, University College London, London, UK; 4 Centre for Astrophysics Research, Department of Physics, Astronomy and Mathematics, University of Hertfordshire, Hatfield, UK; 5 Department of Physics, University of Oxford, Oxford, UK; 6 Center for Astrophysics | Harvard & Smithsonian, Cambridge, MA USA; 7 Department of Astronomy and Astrophysics, University of California, Santa Cruz, Santa Cruz, CA USA; 8 Kavli Institute for Particle Astrophysics and Cosmology (KIPAC), Stanford University, Stanford, CA USA; 9 Department of Physics, Stanford University, Stanford, CA USA; 10 Scuola Normale Superiore, Pisa, Italy; 11 European Space Astronomy Centre (ESAC), European Space Agency (ESA), Madrid, Spain; 12 Centro de Astrobiología (CAB), Spanish National Research Council (CSIC)–National Institute of Aerospace Technology (INTA), Madrid, Spain; 13 Cosmic Dawn Center (DAWN), Copenhagen, Denmark; 14 Niels Bohr Institute, University of Copenhagen, Copenhagen, Denmark; 15 Sorbonne Université, CNRS, UMR 7095, Institut d’Astrophysique de Paris, Paris, France; 16 European Southern Observatory, Garching bei Muenchen, Germany; 17 Max-Planck-Institut für Astronomie, Heidelberg, Germany; 18 Department of Astronomy, University of Wisconsin-Madison, Madison, WI USA; 19 European Space Agency (ESA) Office, Space Telescope Science Institute (STScI), Baltimore, MD USA; 20 Astrophysics Research Institute, Liverpool John Moores University, Liverpool, UK; 21 NRC Herzberg, Victoria, British Columbia Canada; 22 Steward Observatory, University of Arizona, Tucson, AZ USA; 23 Department of Astronomy, University of Texas at Austin, Austin, TX USA; 24 Department of Physics and Astronomy, The Johns Hopkins University, Baltimore, MD USA; 25 NSF’s National Optical-Infrared Astronomy Research Laboratory (NOIRLab), Tucson, AZ USA; 26 School of Physics, University of Melbourne, Parkville, Victoria Australia; 27 AURA for European Space Agency, Space Telescope Science Institute, Baltimore, MD USA; 28 Department of Astrophysical and Planetary Sciences, University of Colorado Boulder, Boulder, CO USA

**Keywords:** Early universe, Galaxies and clusters

## Abstract

Local and low-redshift (*z* < 3) galaxies are known to broadly follow a bimodal distribution: actively star-forming galaxies with relatively stable star-formation rates and passive systems. These two populations are connected by galaxies in relatively slow transition. By contrast, theory predicts that star formation was stochastic at early cosmic times and in low-mass systems^1–4^. These galaxies transitioned rapidly between starburst episodes and phases of suppressed star formation, potentially even causing temporary quiescence—so-called mini-quenching events^5,6^. However, the regime of star-formation burstiness is observationally highly unconstrained. Directly observing mini-quenched galaxies in the primordial Universe is therefore of utmost importance to constrain models of galaxy formation and transformation^7,8^. Early quenched galaxies have been identified out to redshift *z* < 5 (refs. ^9–12^) and these are all found to be massive (*M*_⋆_ > 10^10^ *M*_⊙_) and relatively old. Here we report a (mini-)quenched galaxy at *z* = 7.3, when the Universe was only 700 Myr old. The JWST/NIRSpec spectrum is very blue (*U*–*V* = 0.16 ± 0.03 mag) but exhibits a Balmer break and no nebular emission lines. The galaxy experienced a short starburst followed by rapid quenching; its stellar mass (4–6 × 10^8^ *M*_⊙_) falls in a range that is sensitive to various feedback mechanisms, which can result in perhaps only temporary quenching.

## Abstract (web summary)

Analysis of the JWST/NIRSpec spectrum of the recently observed Lyman-break galaxy JADES-GS+53.15508-27.80178 revealed a redshift of *z* = 7.3, a Balmer break and a complete absence of nebular emission lines, indicating that quenching occurred only 700 million years after the Big Bang.

## Main

The galaxy was first described as a Lyman-break galaxy^13^ and was recently observed as part of our JWST Advanced Deep Extragalactic Survey (JADES; galaxy ID: JADES-GS+53.15508-27.80178; hereafter simply JADES-GS-z7-01-QU) through deep (28-h) NIRSpec-MSA observations with the prism. The galaxy was pre-selected with the photometric Lyman dropout technique and a blue rest-frame ultraviolet (UV) colour.

**Fig. 1:**
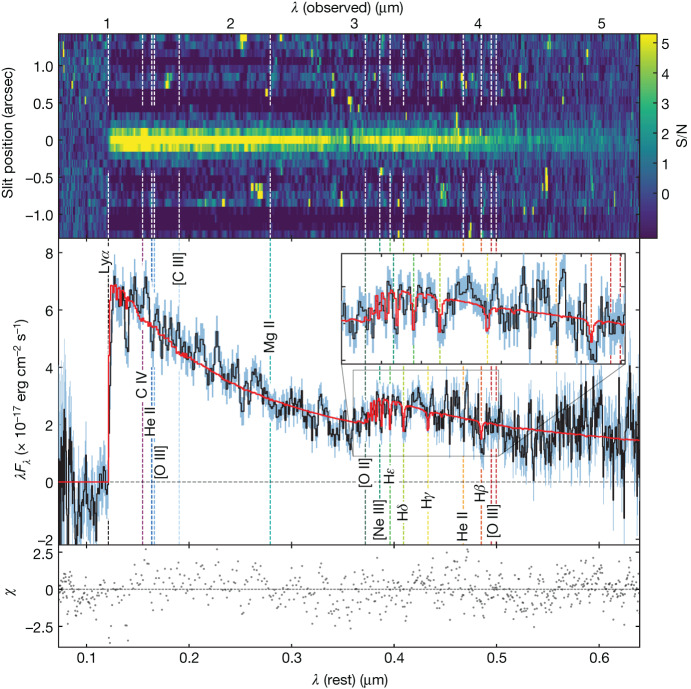
NIRSpec R100/prism spectrum of JADES-GS-z7-01-QU. The absence of emission lines, together with the Balmer break, reveals that this is a—temporarily or permanently—(mini-)quenched, post-starburst galaxy. The clearly detected Ly*α* drop and the Balmer break unambiguously give a redshift of *z* = 7.3. The vertical dashed lines indicate the rest-frame wavelengths of the strongest nebular emission lines. The red line indicates the pPXF spectral fit. The upper panel shows the signal-to-noise ratio (S/N) in the 2D prism spectrum. The bottom panel shows the ratio between the residuals of the fit and the noise. For reference, the flux in the F200W NIRCam filter is 3.33 ± 0.08 × 10^−17^ erg cm^−2^ s^−1^, fully consistent with the spectrum.

The spectrum of JADES-GS-z7-01-QU is shown in Fig. 1. The redshift *z* = 7.29 ± 0.01 is unambiguously determined (using the BEAGLE code; see Methods) from the combined observed wavelengths of the characteristic Ly*α* drop and Balmer break.

The 3*σ* upper limit on the H*β* emission-line flux, *F*(H*β*) < 6.1 × 10^−20^ erg cm^−2^ s^−1^, implies an upper limit on the star-formation rate (SFR) of <0.65 *M*_⊙_ yr^−1^ over the past 3–10 Myr (even accounting for dust attenuation; see Methods). Even stronger constraints come from the [O III]*λ*5008 line: we find *F*([O III]*λ*5008) < 6.5 × 10^−20^ erg s^−1^ cm^−2^, which—combined with a conservative assumption on the [O III]*λ*5008/H*β* ratios in high-*z* galaxies^14,15^—implies a 3*σ* limit on the SFR five times lower than the H*β*-derived value. The absence of emission lines is independently confirmed by the medium-resolution spectrum (see Methods).

We measure a UV slope *β* = −2.09 ± 0.09, typical for galaxies at 6 < *z* < 10 (refs. ^16,17^), indicating strong star-formation activity during the past 100 Myr before observation. In the rest-frame visible, we detect a clear Balmer break and H*δ* absorption with equivalent width ${{\rm{EW}}}_{{\rm{H}}{\delta }_{{\rm{A}}}}=4.8\pm 1.0\,{\text{\AA }}$. This value, combined with the absence of emission lines, means that JADES-GS-z7-01-QU meets the most common spectroscopic definition of a post-starburst galaxy^18,19^, that is, a galaxy that has only recently stopped forming stars.

Previous high-redshift works have identified Balmer-break galaxies in the epoch of reionization^20–22^, indicating the existence of evolved stellar populations and even proposing quiescent phases in these objects^20,21^. However, without spectroscopy, one cannot rule out the presence of emission lines with low equivalent width or that strong emission lines masquerade as Balmer breaks. Furthermore, because of the lack of atmospheric transmission at wavelengths longer than 2.5 microns, it is impossible to investigate Balmer breaks at *z* > 5 from the ground. Therefore, before JWST, it was impossible to confirm the absence of continuing star formation.

Crucially, on the basis of colours alone, this (mini-)quenched galaxy would have been identified as ‘star forming’ by the colour selection criteria^23^, even if including the extension to fast-quenched galaxies^24^. Indeed, its rest-frame *U*–*V* colour of 0.16 ± 0.03 mag places it outside the quiescent region of the *UVJ* diagram, regardless of *V*–*J* colour^25^, as it is the case for other quiescent galaxies at high redshift^7^. However, thanks to JWST/NIRSpec, we can place stringent upper limits on the nebular emission-line fluxes.

Are there potential alternatives to the quenched interpretation? A very high escape fraction of ionizing Lyman-continuum (LyC) photons with *f*_esc_ > 0.9 could strongly suppress nebular emission^26^. However, if *f*_esc_ is high, this would be because nearly all of the interstellar medium (ISM) was ejected or consumed by star formation^27^; yet, if the ISM is absent, there is no fuel for star formation and the galaxy must be quenched. This makes the galaxy highly interesting in the context of reionization, as a remnant leaker^28^. The question is whether the object is (still) a remnant leaker at the epoch of observation. In other words, whether there are still very young stellar populations (a few Myr old) that would still be producing ionizing photons associated with O-type stars and which would largely escape the galaxy, as *f*_esc_ ≈ 1. This scenario is disfavoured by the normal UV slope *β* (ref. ^29^), the Balmer break and by the strong H*δ* absorption.

Statistically, a very recently (<10 Myr) star-forming solution with high *f*_esc_ is also disfavoured by our further analysis. Indeed, by making use of the flexibility of the software BEAGLE to model the observed spectrum, we find that a high-*f*_esc_, recently star-forming solution—although possible—is strongly disfavoured compared with the quenched (>3–10 Myr) solution (see Methods). Furthermore, as we will discuss below, both the pPXF and Prospector codes, which can optionally decouple the continuum from the nebular lines (which are degenerate with *f*_esc_), do not favour a solution with very recent star formation. The second alternative that we cannot completely rule out is the presence of completely obscured star formation, as advocated for some post-starburst galaxies in the local Universe^30^. However, we note that high dust masses and high dust extinction in such low-mass systems, at such high redshift, have never been observed^31^.

To estimate the physical properties of the galaxy including stellar mass *M*_⋆_, SFR, star-formation history (SFH), dust attenuation and stellar metallicity, we apply joint spectrophotometric modelling of its spectral energy distribution (SED). To marginalize over model assumptions and implementation, we use four different SED-fitting codes (pPXF, BAGPIPES, Prospector and BEAGLE; see Methods). Figure 1 shows, as an example, the best-fit pPXF model in red, overlaid on the spectrum.

The methods agree on a low stellar mass of *M*_⋆_ = 4–6 × 10^8^ *M*_⊙_ (Table 1); in other words, this is an object in the dwarf-galaxy regime—essentially the same mass as the nearby, actively star-forming Small Magellanic Cloud, but at *z* = 7.3 and quenched.

**Table 1:** Key physical quantities inferred by the four full spectral fitting codes pPXF, BAGPIPES, BEAGLE and Prospector

Key inferred properties	pPXF	BAGPIPES	BEAGLE	Prospector
log_10_(*M*_⋆_/*M*_⊙_)	–	8.5 ± 0.1	${8.8}_{-0.2}^{+0.1}$	${8.7}_{-0.1}^{+0.1}$
log_10_[SFR (*M*_⊙_ yr^−1^)]	–	<−1.0	$-{2.5}_{-1.0}^{+1.0}$	$-{2.6}_{-2.7}^{+1.5}$
log_10_(*Z*/*Z*_⊙_)	<−2.0	−0.7 ± 0.1	$-{1.9}_{-0.2}^{+0.4}$	$-{1.7}_{-0.2}^{+0.2}$
*t*_quench_ (Myr)	About 50	$1{8}_{-5}^{+5}$	$1{6}_{-4}^{+7}$	$3{8}_{-10}^{+9}$
*t*_form_ (Myr)	About 150	$3{7}_{-5}^{+8}$	$9{3}_{-47}^{+69}$	$11{6}_{-45}^{+85}$
*A*_V_ (mag)	0.4 ± 0.1	$0.3{2}_{-0.23}^{+0.26}$	$0.5{1}_{-0.04}^{+0.03}$	${0.1}_{-0.0}^{+0.1}$

*M*_⋆_, stellar mass; SFR, star-formation rate; *Z*, metallicity; *t*_quench_, quenching lookback time; *t*_form_, formation lookback time; *A*_V_, effective dust attenuation optical depth.

**Fig. 2:**
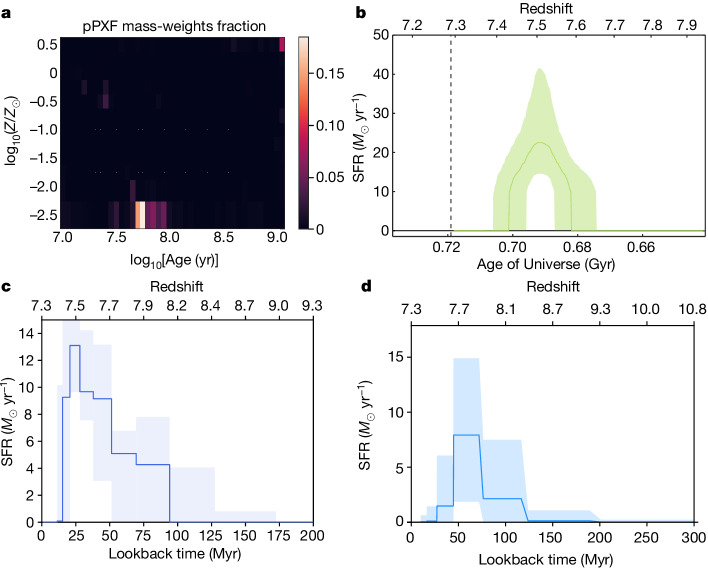
The SFH of the galaxy as inferred by four different full spectral fitting codes, which use different (effective) priors on the SFH of the galaxy. All four codes confirm that the galaxy is quenched at the epoch of observation and reconstruct comparable SFHs. **a**, The stellar age–metallicity grid resulting from the pPXF fit. The code reconstructs dominant metal-poor populations forming from approximately 100 Myr to approximately 20 Myr before observation. The colour bar represents the fractional mass distribution over the SSP grid. **b**, The SFH inferred by BAGPIPES. The solid green line shows the median posterior, the shaded region shows the 1*σ* range, indicating a single star-formation burst lasting approximately 20 Myr and quenching approximately 20 Myr before observation. **c**, The SFH inferred by BEAGLE, which suggests that the galaxy formed approximately 100 Myr before the epoch of observation and quenched approximately 10–20 Myr before observation. **d**, The SFH inferred by Prospector, which suggests that the galaxy quenched approximately 20–30 Myr before observation after a starburst lasting approximately 50 Myr.

Figure 2 shows the SFH of the galaxy, as inferred by the four codes. All models agree that JADES-GS-z7-01-QU is quenched and give similar stellar population parameters. The oldest notable population of stars is 40–150 Myr old, corresponding to a formation redshift *z* = 7.6–8.8, whereas the youngest stars have ages 20–50 Myr, corresponding to a quenching redshift of *z* = 7.4–7.7. These numbers imply that JADES-GS-z7-01-QU formed in a burst of star formation lasting only 20–100 Myr, consistent with the formation timescales of star-forming galaxies at similar redshifts^2^.

The SFR at the time of observation inferred by BAGPIPES, BEAGLE and Prospector are extremely low, between 10^−2.6^ and 10^−1.4^ *M*_⊙_ yr^−1^, yielding specific SFRs ranging between 10^−2.3^ Gyr^−1^ and 0.1 Gyr^−1^. These values are between 2 and 3 orders of magnitude below the main sequence of star-forming galaxies at this redshift^32–36^ and below the widely used threshold sSFR_10_ < 0.2/*t*_H_ = 0.29 Gyr^−1^, on 10-Myr timescales, hence confirming that the galaxy is quenched at the epoch of observation. Crucially, the four codes agree that the galaxy has been strongly star forming between 10 and 100 Myr before the epoch of observation.

Three of the four codes infer a tentative low average stellar metallicity of the galaxy of log_10_(*Z*/*Z*_⊙_) ≈ −2 (in which *Z*_⊙_ is the solar metallicity), whereas BAGPIPES infers log_10_(*Z*/*Z*_⊙_) ≈ −0.7. pPXF indicates the presence of a weak enriched population representing only 5% of the total stellar mass of the galaxy, which formed last before quenching. However, we note that stellar metallicity measurements are uncertain with the low-resolution prism spectroscopy.

Which physical mechanism(s) quenched the galaxy?

The inferred mass of this galaxy rules out that it has been quenched by the UV background^37^; indeed, numerical simulations predict that this quenching mechanism works only for very-low-mass galaxies with *M*_⋆_ ≈ 10^5^–10^7^ *M*_⊙_ (maximally < 10^8^ *M*_⊙_)^38^.

In the local Universe, galaxies in the mass range of our target are quenched primarily by environment^39,40^. It has been postulated that some satellite galaxies may experience environment-driven quenching already during the epoch of reionization^41^. However, we do not find any massive galaxies nearby (see Methods), disfavouring environmental effects as the quenching mechanism for this target.

Given the short inferred duration of the SFH and the rapidity of the transition to quiescence, it seems more reasonable to speculate that JADES-GS-z7-01-QU may have experienced a powerful outflow, driven by either star-formation feedback (radiation-pressure, supernovae might act too slowly) or accretion on a primeval supermassive black hole, which rapidly ejected most of the star-forming gas^42^. This scenario is supported by the tentative low average stellar metallicity inferred by three of the codes. Indeed, ejective feedback mechanisms might have rapidly removed gas from the galaxy and quenched it, before the ISM could be substantially enriched with new metals. A slower quenching process (such as the starvation scenarios) would have probably resulted in a longer transition between star forming and quenched and into higher-metallicity stellar populations, formed out of recycled gas produced by stellar evolution and returned to the ISM by means of supernovae^43,44^.

These outflow events, driven by either star formation or active galactic nucleus, might have mini-quenched star formation only temporarily^45^, until new or re-accreted material replenishes the supply of gas available for star formation and rejuvenates the galaxy. The latter picture may be qualitatively in agreement with a wide range of cosmological simulations predicting that a population of galaxies in the early Universe goes through periodic bursts of star formation, interspersed with periods of suppressed star formation^45–47^. Although the expected SFHs are very ‘bursty’, these recent simulations struggle to achieve the complete quenching observed by us for galaxies with mass similar to our system.

More generally, interpreting these observations with existing simulations is complicated because, according to current theories^47^, this object occupies the transition region between bursty and stable SFHs. Moreover, it is important to note that these models do not include active galactic nucleus feedback, which recent observations have shown to be important in local galaxies of this mass range^48^. These difficulties mean that JADES-GS-z7-01-QU provides the community with the opportunity to shed light on this pivotal mass range.

We conclude by emphasizing that the discovery and spectroscopic analysis of a (mini-)quenched galaxy at redshift *z* = 7.3 by our JADES collaboration ushers the era in which we can constrain theoretical feedback models using direct observations of the primordial Universe. However, this is just the starting point for the JWST mission: upcoming and future observations will start the transition from the ‘discovery’ phase to the statistical characterization of the properties of the first (mini-)quenched galaxies.

## Methods

### JWST/NIRSpec spectra

The NIRSpec^49^ prism/R100 and gratings/R1000 spectra of JADES-GS-z7-01-QU presented in this work were obtained as part of our JADES GTO programme (PI: N. Lützgendorf, ID: 1210) observations in the Great Observatories Origins Deep Survey South (GOODS-S) field between 21 and 25 October 2022. The R100 observations were obtained using the disperser/filter configuration PRISM/CLEAR, which covers the wavelength range between 0.6 μm and 5.3 μm and provides spectra with a wavelength-dependent spectral resolution of *R* ≈ 30–330. The R100 spectrum of JADES-GS-z7-01-QU is presented in Fig. 1.

**Extended Data Fig. 1:**
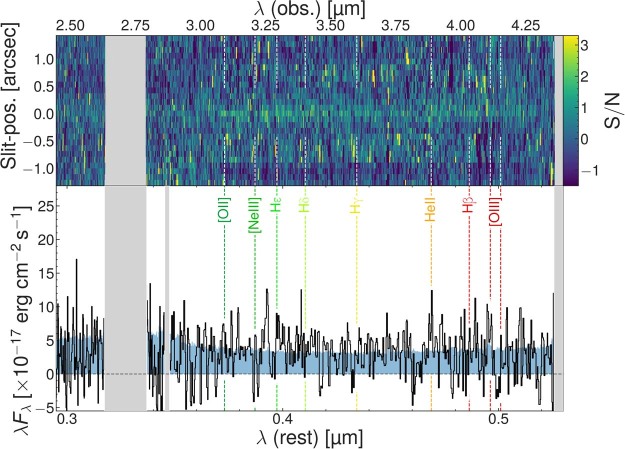
NIRSpec R1000/grating spectrum of the (mini-)quenched galaxy JADES-GS-z7-01-QU at *z* = 7.3. The spectrum confirms the absence of emission lines. The blue-shaded region shows the 1D noise level. The upper panel shows the signal-to-noise ratio (S/N) in the 2D grating spectrum. The spectrum is median-smoothed, for visualization.

The medium-resolution R1000 observations, with a spectral resolution of *R* ≈ 500–1,340, used the disperser/filter configurations G140M/F070LP, G235M/F170LP and G395M/F290LP, which were exposed for 14 h, 7 h and 7 h. A zoom-in on the R1000 spectrum (into the region with spectral lines best tracing star-formation activity) is shown in Extended Data Fig. 1. Finally, high-resolution R2700 observations used G395H/F290LP and were exposed for 7 h (like the R1000 spectrum, the R2700 spectrum of JADES-GS-z7-01-QU contains no detections, hence is not shown).

The programme observed a total of 253 galaxies over three dither pointings, with JADES-GS-z7-01-QU being observed in each of the three pointings. Each dither pointing had a different microshutter array (MSA) configuration to place the spectra at different positions on the detector to decrease the impact of detector gaps, mitigate detector artefacts and improve the signal-to-noise ratio for high-priority targets, while increasing the density of observed targets. Within each individual dither pointing, the telescope executed a three-nod pattern (by slightly reorienting the telescope by the length of one microshutter, keeping the same MSA configuration). In each of the three nodding pointings, three microshutters were opened for each target, with the targets in the central shutter. Each three-point nodding was executed within 8,403 s. The nodding pattern has been repeated four times in the PRISM/CLEAR configuration, two times in the G140M/F070LP combination, once in the G235M/F170LP combination and once in the G395M/F290LP combination. This resulted in a total exposure time for JADES-GS-z7-01-QU of 28 h in R100, 14 h in G140M and 7 h in each of G235M, G395M and G395H.

The flux-calibrated spectra were extracted using a customized pipeline developed by the NIRSpec GTO team, which builds on the publicly available ESA NIRSpec Science Operations Team (SOT) pipeline^50^. A detailed description of the custom pipeline will be presented in a forthcoming technical paper (Carniani et al., in preparation) and more information can be found in ref. ^51^. We summarize here the main steps and the differences to the publicly available pipeline. For each exposure, we extract the count rate for each pixel, removing cosmic rays and flagging saturation. The 2D spectrum is background subtracted on the basis of the two other exposures in the three-nod pattern. The individual 2D spectra are flat-fielded and illumination-corrected, taking into account the wavelength-dependent throughput. The wavelength and flux calibration was then applied, with each pixel of the 2D spectrum having an associated wavelength and position along the shutter. We applied a wavelength-dependant path-loss correction to account for flux falling outside the microshutter, taking into account the considerable point spread function variation of NIRSpec, treating the target as a point source. For the prism, we used an irregular spectral wavelength grid, taking into account the resolution (*R*) as a function of wavelength. The 1D spectra for the three nod positions from each of the three pointings are combined by a weighted average into a single 1D spectrum. Outliers are rejected with a sigma-clipping algorithm. The presented 1D spectra come from a combination of the 1D individual spectra and are not an extraction from the presented combined 2D spectra.

### JWST/NIRCam image and morphology

**Extended Data Fig. 2:**
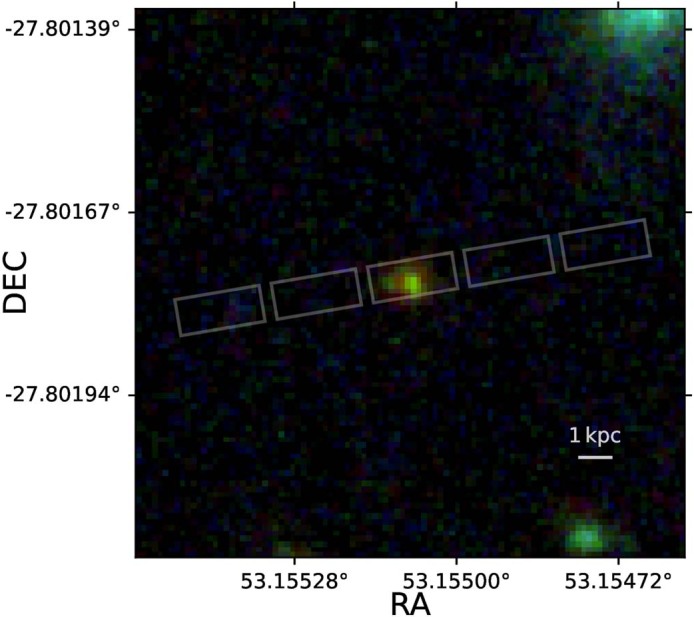
JWST/NIRCam image covering JADES-GS-z7-01-QU and its nearby projected environment. The NIRCam F444W-F200W-F090W rgb colour image is created from cutouts of the mosaics at wavelengths *λ* ≈ 0.8–5 μm. The five NIRSpec microshutter positions used for this target are overlaid in white.

A JWST/NIRCam F444W-F200W-F090W rgb (red-green-blue) colour image of JADES-GS-z7-01-QU from our JADES programme (PI: Daniel J. Eisenstein, ID: 1180), created from cutouts of the mosaics in each filter, at wavelengths *λ* ≈ 0.8–5 μm, is shown in Extended Data Fig. 2.

For the spectrophotometric modelling of JADES-GS-z7-01-QU, we used the photometry from the JADES and JEMS^52^ NIRCam^53,54^ surveys. In particular, the modelling included deep infrared NIRCam observations with the following filters: F090W, F115W, F150W, F182M, F200W, F210M, F277W, F335M, F356W, F410M, F430M, F444W, F460M and F480M. The JADES photometry reduction pipeline made use of the JWST Calibration Pipeline (JWSTCP, v1.9.2) with the CRDS pmap context 1039. The raw images were transformed into count-rate images, making use of JWSTCP stage 1, for which detector-level corrections and ‘snowballs’ were accounted. The count-rate images were then flat-fielded and flux-calibrated with a customized methodology, using JWSTCP stage 2. Finally, the mosaics were created using stage 3 of the pipeline. For further details on the JADES photometry data reduction pipeline, see refs. ^55,56^.

To obtain the morphological parameters of JADES-GS-z7-01-QU, we fit the NIRCam photometry with Forcepho (Johnson et al., in preparation). Forcepho models galaxies and substructures (for example, clumps or blended companions) as several Sérsic profiles convolved with the instrument point spread functions as mixtures of Gaussians by forward-modelling the light distribution in all individual exposures and filters and sampling the joint posterior probability distribution of all parameters through Markov chain Monte Carlo. For more details on the multicomponent modelling procedure, see ref. ^56^. JADES-GS-z7-01-QU appears as a compact, discy galaxy (half-light radius *R*_e_ = 36 ± 1 mas ≙ 0.2 kpc ≙ 0.04 arcsec, Sérsic index *n* = 0.95 ± 0.03; Extended Data Fig. 2). The images also show a distinct, fainter source 0.13 arcsec to the east. This secondary source could not be deblended in the spectroscopy but we obtained deblended photometry using Forcepho. The contribution of the secondary source to the total flux ranges from a maximum of 27% (in the F115W band) to 17% (in the F444W band), therefore its SED is much bluer than that of the main source. Its photometric redshift *z* = 7.50 ± 0.13 (1*σ*) is consistent with the spectroscopic redshift of the main source. At a redshift of *z* = 7.3, this secondary source would lie within 0.7 kpc (or 3*R*_e_) from the centre of JADES-GS-z7-01-QU; its interpretation as a clump or satellite is unclear. To attempt removing its contribution from the spectrum of the main source, we extracted a spectrum from the central three pixels (0.3 arcsec) from the NIRSpec 2-d spectrum; using this spectrum does not change the interpretation of our results, that is, JADES-GS-z7-01-QU is still quenched.

As discussed in the main text, quenching by environment is ruled out for JADES-GS-z7-01-QU, as no other galaxy resides nearby. This can be verified with JADES NIRCam imaging on our publicly available website and more specifically the interactive tool FitsMap: https://jades.idies.jhu.edu/public/?ra=53.1554497&dec=-27.8018917&zoom=9 at the coordinates RA = 53.1551 and dec. = −27.8018.

### Full spectral fitting

#### pPXF

The red model fit of the stellar continuum in Fig. 1 was performed with the *χ*^2^-minimization Penalized PiXel-Fitting code pPXF (refs. ^57,58^), using a library of single stellar population (SSP) templates spectra obtained combining the synthetic C3K model atmospheres^59^ with MIST isochrones^60^ and solar abundances. The SSP spectra span a full 2D logarithmic grid of 62 ages and 10 metallicities from age_SSP_ = 10^6.0^ years to 10^9.2^ years (generously older than the age of the Universe at *z* = 7.3) and log_10_(*Z*/*Z*_⊙_)_SSP_ = −2.5 to 0.5. Owing to the low resolution of the R100 spectrum, we fix the stellar velocity dispersion to its virial estimate ${\sigma }_{* }\approx {\sigma }_{{\rm{vir}}}\equiv \sqrt{{{\rm{GM}}}_{* }/(5{R}_{{\rm{e}}})}=50\,{\rm{km}}\,{{\rm{s}}}^{-1}$. To account for dust reddening, the fitted SSP are multiplicatively coupled to the dust attenuation curve in ref. ^61^. To infer the stellar population weight-grid shown in Fig. 2a, following ref. ^62^, we first convolve the SSP templates to match the wavelength-dependant spectral resolution of the prism spectrum. Then, to avoid numerical problems, both the spectrum and the templates are renormalized by the median flux per spectral pixel. Then we run an initial fit with pPXF and we *σ*-clip outliers in the spectrum. Finally, we perform a residual-based bootstrapping of the initial pPXF best fit, without regularization^57,58^, over 1,000 iterations. The inferred bootstrapped SSP grids are averaged to recover the non-parametric SFH, consistent with the intrinsic noise of the spectrum, presented in Fig. 2a.

We infer a dust attenuation of the stars in this galaxy of *A*_V_ = 0.4 ± 0.1. It should be noted that the presence of dust in the pPXF fit is mainly driven by the UV slope. The complex physics of the Ly*α* drop is not included in the SSP templates. Masking this part of the spectrum returns a nearly dust-free fit with older and metal-richer stellar populations, which would make JADES-GS-z7-01-QU even more quenched. As stated in the main text, we infer an extremely low average stellar metallicity of log_10_(*Z*/*Z*_⊙_) ≈ −2 with pPXF. It should be noted that the dominant reconstructed stellar populations lie at log_10_(*Z*/*Z*_⊙_) ≈ −2.5, at the boundary of the available grid of synthetic spectra. This suggests that model SSP spectra of even lower metallicity might be needed in the future to accurately model the stellar populations in galaxies at high redshift. However, we note that the metallicity measurements are uncertain, owing to the low resolution of the prism. We infer that the oldest notable population of stars (that is, indicating the start of the star formation) in the galaxy is 150 Myr old, whereas the youngest is 50 Myr old, resulting in an extremely short duration of the star formation of just 100 Myr between the formation of the galaxy and its quenching.

#### BAGPIPES

**Extended Data Fig. 3:**
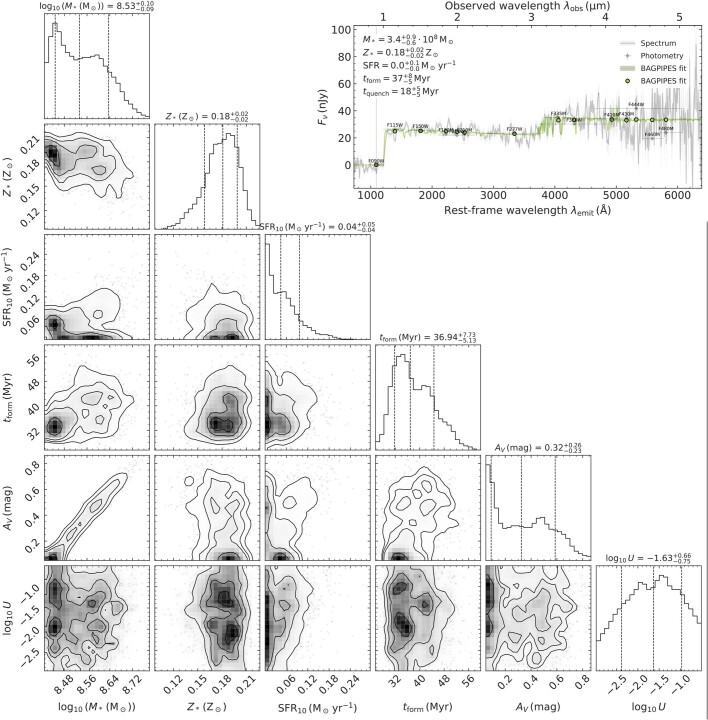
Summary of key outputs by BAGPIPES. Bottom left, corner plot. Top right, spectrophotometric BAGPIPES fit of the JADES-GS-z7-01-QU R100/prism spectrum.

We used the Bayesian Analysis of Galaxies for Physical Inference and Parameter EStimation (BAGPIPES) code^63^ to simultaneously fit the NIRSpec PRISM measurements and NIRCam photometry. Following ref. ^64^, we used the updated BC03 stellar population models^65,66^ combined with the stellar MILES library^67^ and the updated stellar evolution tracks^68,69^. For the presented BAGPIPES fit, we assumed two bins of constant SFH, one fixed bin over the past 10 Myr and one variable bin spanning a range beyond 10 Myr (minimum age ranging between 10 Myr and 0.5 Gyr, maximum age between 11 Myr and the age of the Universe). We varied the total stellar mass formed between 0 and 10^15^ *M*_⊙_ and the stellar metallicity of the variable SFH bin between 0.01 *Z*_⊙_ and 1.5 *Z*_⊙_ (the 10-Myr bin having a metallicity of 0.2 *Z*_⊙_ to match the inferred metallicity of the variable-SFH bin). Nebular emission is modelled self-consistently with a grid of CLOUDY^70^ models with the ionization parameter (−3 < log_10_*U* < −0.5) as a free parameter. We included a flexible dust attenuation prescription^71^ with visual extinction and power-law slope freely varying (0 < *A*_V_ < 7, 0.4 < *n* < 1.5) while fixing the fraction of attenuation from stellar birth clouds to 60% (the remaining fraction arising in the diffuse ISM; ref. ^72^). A first-order correction polynomial^73^ is fitted to the spectroscopic data to account for aperture and flux calibration effects. The spectrophotometric fit and the corresponding corner plot are shown in Extended Data Fig. 3. We find that nearly no wavelength-dependant correction is necessary at the blue end of the spectrum, whereas at the red end, a correction of 15% is applied. Crucially, we find a very low SFR (consistent with 0) in the past 10 Myr for JADES-GS-z7-01-QU, noting that other tested SFH parametrizations, namely the double-power-law SFH described in ref. ^74^ and a single-bin constant SFH with flexible beginning and end of star formation, return consistent results and most crucially agree that the galaxy is quenched. We infer that the oldest stellar population is 40 Myr old, which is equivalent to a formation redshift of *z* = 7.6. The galaxy has been quenched for 10 Myr, resulting in a short duration of star formation of 20 Myr from the formation of the galaxy to its quenching.

#### BEAGLE

We use the Bayesian analysis tool BEAGLE (ref. ^66^) to fit to the R100/prism spectrum of JADES-GS-z7-01-QU. The BEAGLE code incorporates a consistent modelling of stellar radiation and its transfer through the interstellar and intergalactic media. We model the SFH as an initial delayed exponential with maximum stellar age, *t*_form_ (years), and location of the peak of star formation as free parameters. To disentangle the current SFR from the integrated property of total stellar mass, we allow for the most recent episode of star formation to be modelled as a constant with free parameters SFR (*M*_⊙_ yr^−1^) and duration, *t*_quench_ (years) (which can vary between 10^7^ and 10^8^ years). The nebular emission is characterized by the interstellar metallicity, the ionization parameter, the mass fraction of interstellar metals locked within dust grains and, crucially, *f*_esc_ (which can vary between 0 and 1). Dust attenuation follows the two-component prescription of ref. ^71^, in which we fit for the total effective V-band attenuation optical depth (fixing the ratio of V-band ISM attenuation to the V-band ISM + birth cloud attenuation to 0.4). We also fit for stellar metallicity, stellar mass formed and redshift, totalling 12 free parameters. A list of the free parameters and the adopted priors is presented in Extended Data Table 1.

**Extended Data Fig. 4:**
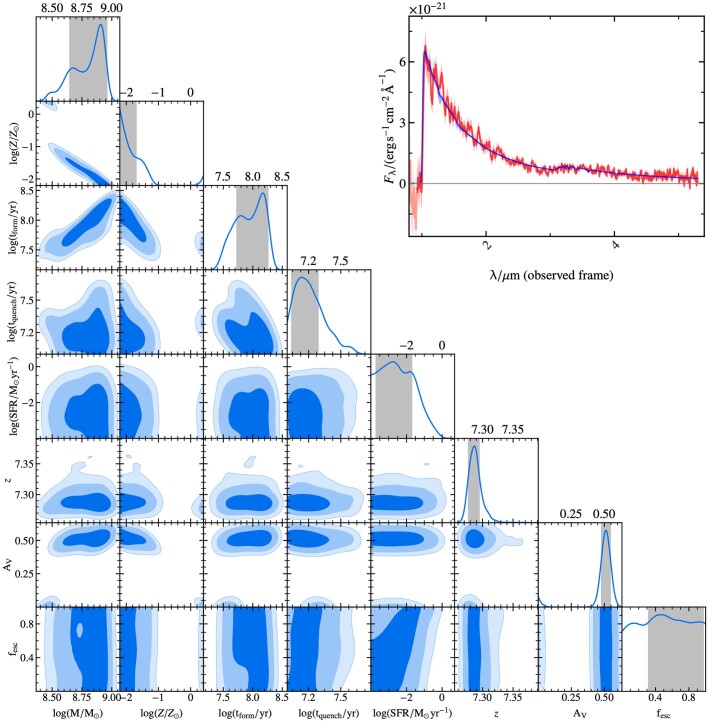
Summary of key outputs by BEAGLE. Bottom left, corner plot with free *f*_esc_. Top right, BEAGLE maximum a posteriori model of the R100 spectrum.

The corner plot in Extended Data Fig. 4 shows the BEAGLE posterior probability distributions of the BEAGLE fit. The 2D (off-diagonal) and 1D (along the main diagonal) subplots show the posterior distributions on stellar mass *M*_⋆_, metallicity *Z*, SFR, maximum age of stars *t*_form_, minimum age of stars *t*_quench_, redshift *z*, effective dust attenuation optical depth in the V-band *A*_V_ and the escape fraction of ionizing photons *f*_esc_. The dark, medium and light blue contours show the extents of the 1*σ*, 2*σ* and 3*σ* credible regions.

BEAGLE gives a current SFR of less than 10^−1.5^ *M*_⊙_ yr^−1^, a formation time of less than 160 Myr before observation and a quenching time of roughly 15 Myr before observation.

We also note that BEAGLE, as for the other three codes, requires some degree of dust attenuation, which suggests that some cold gas is still present, which—in turn—is incompatible with *f*_esc_ ≈ 1.

#### Prospector

**Extended Data Fig. 5:**
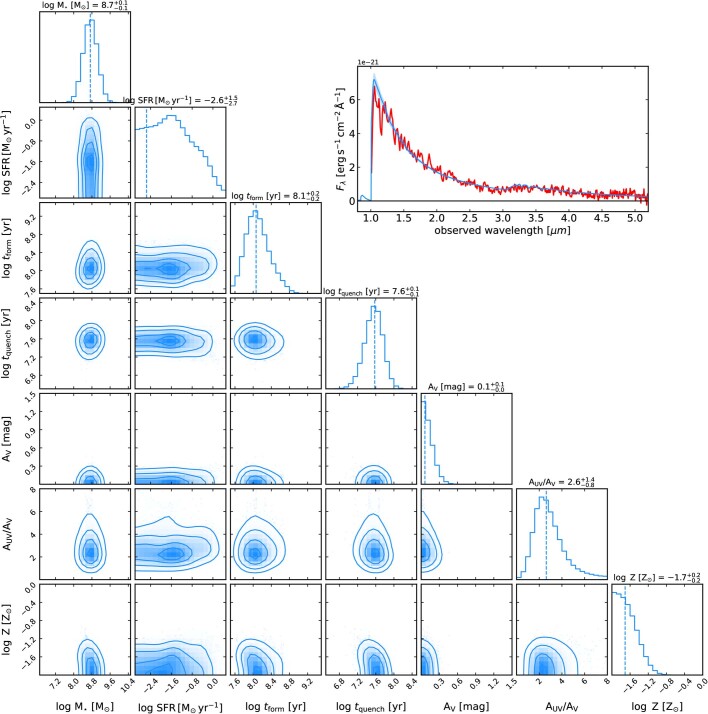
Summary of key outputs by Prospector. Bottom left, corner plot with stellar mass *M*_⋆_, SFR, *t*_form_, *t*_quench_, dust attenuation *A*_V_, *A*_UV_/*A*_V_ and stellar metallicity *Z*. Top right, Prospector maximum a posteriori model of the R100 spectrum.

We use the Bayesian SED fitting code Prospector^75^ to model the spectrophotometric data of JADES-GS-z7-01-QU. The posterior corner plot for several key parameters from Prospector is shown in Extended Data Fig. 5. The code uses a flexible spectroscopic calibration model, combined with forward modelling of spectra and photometry, to infer physical properties. Following the setup in ref. ^76^, we include a flexible SFH (ten bins with the bursty continuity prior), a flexible attenuation law (diffuse dust optical depth with a power-law modifier to shape the attenuation curve of the diffuse dust in ref. ^61^) and fit for the stellar metallicity. Notably, Prospector infers a low-dust attenuation with ${A}_{{\rm{V}}}={0.1}_{-0.0}^{+0.1}$ with a rather steep attenuation law $\left({A}_{{\rm{UV}}}/{A}_{{\rm{V}}}={2.6}_{-0.8}^{+1.4}\right)$. This is consistent with the idea that the galaxy has a low gas content and the low SFR in the past 30 Myr before observation. Prospector infers that the oldest stellar population (as defined by the lookback time when the first 10% of the stellar mass formed) has an age of about 100 Myr, which means a nominal formation redshift of *z* = 8.8. The SFR increases markedly approximately 80 Myr before observation. After this final burst, lasting around 50 Myr, the galaxy quenched on a short timescale.

We have also experimented with the standard continuity prior^77^, which weights against sharp transition in the SFH. The overall shape of the SFH is the same, indicating that the data strongly prefer a decreasing SFH in the past roughly 50 Myr. Quantitatively, the recent SFR (averaged over the past 10 Myr) increases with this prior to ${\log }_{10}\left({\rm{SFR}}\,\left({M}_{\odot }\,{{\rm{year}}}^{-1}\right)\right)=-{0.4}_{-0.9}^{+0.4}$, which is still consistent with being quenched and within the uncertainties of the fiducial value obtained with the bursty continuity prior. The quenching time is slightly more recent $\left(2{4}_{-9}^{+6}\,{\rm{Myr}}\right)$, but consistent within the uncertainties quoted in Table 1.

### Star-forming, high-*f*_esc_ interpretation

It should be noted that the complete absence of nebular lines always allows, by construction, a solution with *f*_esc_ ≈ 1 (regardless of whether the galaxy has been recently star forming or quiescent)—the question is whether this solution is accompanied by the production of ionizing photons associated with continuing star formation.

The fiducial BEAGLE posterior distribution does not highlight a solution with high *f*_esc_ and very recent star formation^26,29,78^. By contrast, although *f*_esc_ is unconstrained, even a value approaching unity indicates a low SFR < 0.1 *M*_⊙_ yr^−1^ at the 3*σ* level (fifth subplot from the left at the bottom of Extended Data Fig. 4).

To assess the very recently star-forming and high-*f*_esc_ scenario quantitatively, we use BEAGLE to compare two SED models. The model already described (see the ‘BEAGLE’ section) formally allows a star-forming solution with high *f*_esc_. The alternative model has a simplified SFH consisting of a constant SFR; in this way, low-SFR solutions are effectively removed by the constraint to form sufficient stellar mass of the appropriate age to reproduce the observed spectrum. This alternative model gives ${f}_{{\rm{esc}}}=0.9{8}_{-0.04}^{+0.01}$ and ${\rm{SFR}}=0.6{3}_{-0.05}^{+0.05}\,{M}_{\odot }\,{{\rm{year}}}^{-1}$, which is a much higher SFR than the alternative solution. To select the preferred model, we use the Bayes ratio, that is, the ratio between the evidence of the models. The log difference between the evidences, that is, the Bayes factor, is ln(*K*) = 4.1 ± 0.3; according to Jeffreys’ criterion^79^, this is strong evidence for the quenched solution and we adopt it as our fiducial model.

As an extra test, we assumed a model with the same setup as the fiducial run, but forcing the escape fraction to *f*_esc_ > 0.9. We find that the result is equal to the fiducial run and the galaxy remains quenched.

### Empirical measurements

To estimate the flux upper limits on H*β* and [O III]*λ*5008, we sum the formal variance over three pixels. For ${{\rm{EW}}}_{{\rm{H}}{\delta }_{{\rm{A}}}}$ we use the bands in the Lick definition^80^ but without any further correction owing to spectral resolution.

We derive an upper limit on the SFR from the 3*σ* upper limit on the H*β* emission-line flux, *F*(H*β*) < 6.1 × 10^−20^ erg cm^−2^ s^−1^. To correct this flux for dust attenuation, we assume the Milky Way attenuation law^81^, which seems appropriate for galaxies at least up until *z* = 2.5 (refs. ^82,83^). Given that H*β* is not detected, we cannot measure the Balmer decrement. We therefore derive the nebular *A*_V_ from the continuum *A*_V_ = 0.51 mag inferred from BEAGLE (the highest value between all models) and upscale this value by 0.64, the median continuum-to-nebular *A*_V_ ratio inferred from local galaxies^84^ (of stellar mass comparable with JADES-GS-z7-01-QU). The flux is converted to a luminosity assuming the Planck18 cosmology^85^. To convert the H*β* attenuation-corrected luminosity to a SFR, we use the conversion factor 2.1 × 10^−42^ *M*_⊙_ yr^−1^ erg^−1^ s, appropriate for a Chabrier initial mass function with a high-mass cutoff of 100 *M*_⊙_ and metallicity *Z* = 0.27 *Z*_⊙_ (ref. ^83^) (note that this value of the metallicity is higher than that inferred from the data; this provides a conservative estimate). This gives a SFR of 0.57 *M*_⊙_ yr^−1^. Even stronger constraints come from the [O III]*λ*5008 line: we find *F*([O III]*λ*5008) < 6.5 × 10^−20^ erg s^−1^ cm^−2^, which, combined with a conservative assumption of [O III]*λ*5008/H*β* ratios in high-*z* galaxies^14,15^, implies a 3*σ* limit on the SFR roughly five times lower than the H*β*-derived value (SFR = 0.12 *M*_⊙_ yr^−1^).

Alternatively, assuming the median (and the extreme) observed Balmer decrement 3.5 (5.5) from ref. ^83^, we would obtain nebular *A*_V_ values of 0.63 and 2.05 mag, respectively. These translate into [O III]*λ*5008-derived SFRs of 0.10 and 0.34 *M*_⊙_ yr^−1^, respectively. As a comparison, the SFR threshold for quiescence at *z* = 7.3 is 0.18 *M*_⊙_ yr^−1^ (obtained from the threshold in sSFR defined by 0.2/*t*_H_(*z*) times the BEAGLE stellar mass (ref. ^86^)). Thus, in all but the most extreme scenario, JADES-GS-z7-01-QU would meet the formal threshold for quiescence. The absence of emission lines is independently confirmed by the medium-resolution spectrum (see Extended Data Fig. 1).

## Online content

Any methods, additional references, Nature Portfolio reporting summaries, source data, extended data, supplementary information, acknowledgements, peer review information; details of author contributions and competing interests; and statements of data and code availability are available at 10.1038/s41586-024-07227-0.

## Figures and Tables

**Extended Data Table 1:**
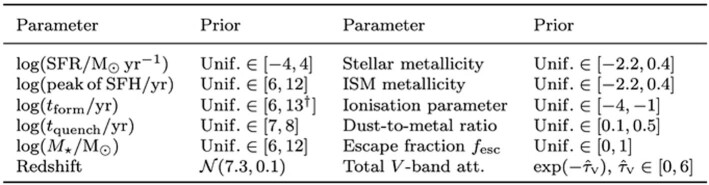
Parameters and associated priors set in BEAGLE

${\mathcal{N}}(a,b)$ is the normal distribution with mean *a* and standard deviation *b*. †In practice, BEAGLE will not allow the age of the oldest stars to be greater than the time between *z* = 20 and the sampled redshift.

## Data Availability

The reduced spectra that support the findings of this study are publicly available on GitHub: https://github.com/tobiaslooser/JWST-reveals-a-recently-mini-quenched-galaxy-at-z-7.3. See MAST at Space Telescope Science Institute for the original data: https://archive.stsci.edu/hlsp/jades.

## Code availability

The pPXF, BAGPIPES and Prospector codes are publicly available. BEAGLE is available through a Docker image (distributed through Docker Hub) on request at https://www.iap.fr/beagle/.
